# 4433 SGRQ score is associated with treatment status for patients with non-tuberculous mycobacterial lung disease

**DOI:** 10.1017/cts.2020.146

**Published:** 2020-07-29

**Authors:** Bryan Garcia, Abigail Grady, Lilian Christon, Patrick Flume, Susan Dorman

**Affiliations:** 1 Medical University of South Carolina; 2 MUSC

## Abstract

OBJECTIVES/GOALS: The Saint Georges Respiratory Questionnaire (SGRQ) is used as a patient reported outcome tool for clinical research in COPD and bronchiectasis. We established a registry and biospecimen repository of bronchiectasis patients with and without NTM and report associations between clinical phenotype and SGRQ scores. METHODS/STUDY POPULATION: Patients were recruited in a cross-sectional format from the Bronchiectasis, Cystic Fibrosis, and NTM clinics at our institution. All patients provided at least one sputum sample in the six months prior to inclusion. Clinical and epidemiologically relevant data was obtained, and blood specimens were processed and preserved. Patients were grouped based on clinical phenotype and differences in SGRQ scores were analyzed using ANOVA or Student’s t-test. Descriptive statistics are reported as means and standard deviations, p<0.05 considered significant. RESULTS/ANTICIPATED RESULTS: 72 NTM patients completed the SGRQ including 39 patients not on treatment (Colonized), 29 patients on NTM directed antibiotics, and 4 patients whose infection was cured in the past year. Among patients on treatment, 14 were treatment refractory (positive cultures beyond 12 months of therapy). The mean age of all NTM patients was 59.5±17.6 and 80.5% were female. Mean SGRQ Total scores were significantly higher among patients receiving treatment compared to patients considered colonized (35.7± 22.0 colonized group versus 48.8± 15.8 treatment group, p = 0.011). The SGRQ subdomain scores including Impacts (26.2± 26.2 colonized group versus 42.5± 17.0 treatment group, p = 0.01) and Activities (41.7± 31.8 colonized group versus 59.3± 24.5 treatment group, p = 0.018) were also significantly different between groups. DISCUSSION/SIGNIFICANCE OF IMPACT: We developed a cross sectional cohort of NTM patients and assessed associations between clinical phenotype and SGRQ score. Preliminary data suggests that female sex, treatment status, and therapeutic duration are associated with higher SGRQ scores. We intend to continue to assess the potential for specific SGRQ questions to be used for quantifying disease symptom severity for NTM patients.

